# Boosting Blue Self-Trapped Exciton Emission in All-Inorganic Zero-Dimensional Metal Halide Cs_2_ZnCl_4_ via Zirconium (IV) Doping

**DOI:** 10.3390/molecules29071651

**Published:** 2024-04-06

**Authors:** Ye Tian, Qilin Wei, Lian Duan, Chengyu Peng

**Affiliations:** 1 School of Semiconductors and Physics, North University of China, Taiyuan 030051, China; 20230127@nuc.edu.cn; 2 School of Chemistry and Chemical Engineering, Shandong University, Jinan 250100, China; 3 Traffic Information Engineering Institute, Guangxi Transport Vocational and Technical College, Nanning 530004, China; duanlianxs@foxmail.com

**Keywords:** low-dimensional metal halides, blue emission, Zr^4+^:Cs_2_ZnCl_4_, self-trapped excitons

## Abstract

Low-dimensional metal halides with efficient luminescence properties have received widespread attention recently. However, nontoxic and stable low-dimensional metal halides with efficient blue emission are rarely reported. We used a solvothermal synthesis method to synthesize tetravalent zirconium ion-doped all-inorganic zero-dimensional Cs_2_ZnCl_4_ for the first time. Bright blue emission in the range of 370 nm–700 nm with a emission maximum at 456 nm was observed in Zr^4+^:Cs_2_ZnCl_4_ accompanied by a large Stokes shift, which was due to self-trapped excitons (STEs) caused by the lattice vibrations of the twisted structure. Simultaneously, the PLQY of Zr^4+^:Cs_2_ZnCl_4_ achieve an impressive 89.67%, positioning it as a compelling contender for future applications in blue-light technology.

## 1. Introduction

The broad prospects of solid-state lighting and full-color displays have promoted the rapid development of luminescent materials. Red, green, and blue light sources covering the entire visible light range are required to produce effective white light. The design and manufacture of blue light has proven to be the most challenging of the three sources. For example, blue light-emitting diodes became available two decades after red and green light-emitting diodes were successfully manufactured using epitaxial inorganic technology [1,2].

In the past decade, low-dimensional metal halide luminescent materials have been extensively studied. In particular, zero-dimensional (0D) metal halides have attracted widespread attention due to their high photoluminescence quantum yields (PLQYs) [3]. Various crystal materials including tetrahedral BX_4_, pyramidal BX_5_, and octahedral BX_6_ have been reported in metal halides with a 0D structure [4,5,6]. Green, yellow, and red emission with high PLQY was achieved in 0D metal halides [5,7,8,9,10]. However, blue-emitting metal halides with high efficiency and stability remain challenging. Recently, some lead-based low-dimensional metal halides with efficient blue-light emission have been reported [11,12,13]. Despite the fascinating optical properties of these Pb-based compounds, the toxicity of Pb^2+^ hinders their further practical applications. Therefore, it is particularly important to design low-toxic or non-toxic low-dimensional metal halide materials with efficient blue emission properties for future applications. At present, Cu(I)-based compounds with high-efficiency blue emission characteristics are the most representative low-toxic halide materials and have received widespread attention from the scientific community [14,15,16,17,18]. The luminescence of Cu(I)-based low-dimensional metal halides comes from the self-trapped excitons formed by its soft lattice structure. The existence of organic macromolecules and large-sized inorganic cations enable the production of a strong spatial confinement effect, resulting in photogenerated excitons. These photogenerated excitons are strongly bound within the inorganic polyhedron which then emits photons outward through radiative recombination, finally producing efficient luminescence. However, the naturally unstable and easily oxidized characteristics of Cu(I) are still a problem that needs to be solved urgently. Xia et al. reported a zero-dimensional Sn(IV)-based organic–inorganic hybrid meatal halide (C_6_N_2_H_16_Cl)_2_SnCl_6_ [19]. This material exhibited broadband blue-light emission, which came from the self-trapped excitons formed inside the crystal. At the same time, (C_6_N_2_H_16_Cl)_2_SnCl_6_ also had ultra-high stability. The PL intensity of (C_6_N_2_H_16_Cl)_2_SnCl_6_ remained stable for more than three months. Its PL intensity remained at about 50% of the initial intensity when the temperature rose to 450 K. However, the emission efficiency of (C_6_N_2_H_16_Cl)_2_SnCl_6_ was only 8.1%, which is too low for large-scale commercial applications. Through theoretical calculations, they confirmed that the lower PLQY of (C_6_N_2_H_16_Cl)_2_SnCl_6_ was caused by the disappearance of the lone-pair electrons on the Sn^4+^ cations, which led to the smallest distortion and finally led to the low PLQY. In addition to Sn(IV)-based compounds, In(III)-based compounds have also attracted much attention recently. Saparov et al. reported a new zero-dimensional organic–inorganic hybrid indium bromide RInBr_4_ [R = trimethyl(4-stilbenyl)methylammonium cation] [20]. The RInBr_4_ showed bright-blue emission at 437 nm under the 391 nm UV excitation with Commission Internationale de l’Eclairage color coordinates of (0.19, 0.20) and a high photoluminescence quantum yield of 16.36% at room temperature. In addition, this novel hybrid indium bromide demonstrated significantly improved environmental stability. In addition to indium bromide, low-dimensional organic–inorganic hybrid indium chloride with blue-light emission has also been reported. Yue et al. reported two indium chlorides with blue-light emission: [H_2_EP]_2_InCl_6_·Cl·H_2_O·C_3_H_6_O and [H_3_AEP]InCl_6_··H_2_O (EP = 1-ethylpiperazine, AEP = N-aminoethyl piperazine) [21]. The blue emission of both compounds came from self-trapped excitons generated by their soft lattice structures. However, the PLQY of [H_2_EP]_2_InCl_6_·Cl·H_2_O·C_3_H_6_O and [H_3_AEP]InCl_6_··H_2_O was not high. The highest was only 13.44%, which is far from meeting the needs of practical applications. A series of zero-dimensional In-based halides: [H_3_AEPz]_2_InCl_9_·(H2O)_2_ (AEPz = N-aminoethylpiperazine), [H_2_AMPd]_2_InCl_7_ [AMPd = 4-(aminomethyl)piperidine], and [H_2_PhPz]_2_InCl_7_·(H2O)_2_ (PhPz = 1-phenylpiperazine), reported by Lei et al., had a maximum PLQY of no more than 20% [22]. Therefore, In(III)- and Sn(IV)-based materials are still plagued by problems such as low luminous efficiency and poor optical properties, which cannot meet the needs of practical applications [19,20,21,22].

In recent years, low-dimensional Zn(II)-based materials based on Zn with a d^10^ electronic configuration have gradually attracted the attention of researchers due to their variable crystal structure, low toxicity, and high stability properties. Currently, efficient emission is achieved in the all-inorganic zero-dimensional Zn-based compound Cs-Zn-X (X = Cl, Br, and I) by doping modification with Cu^+^ ions. Through structural transformation and halogen control, the emission color of Cs-Zn-X can be effectively adjusted from violet to blue [5,15,23,24]. However, these materials still face the problem of poor stability due to the presence of Cu^2+^ ions. After being left for a period of time, their optical performance will gradually deteriorate due to the oxidation of Cu^+^ to Cu^2+^ until they lose optical activity. The key to solving this problem is whether we can find a stable ion as a dopant and incorporate it into the Cs-Zn-X crystal lattice so that it can produce efficient and stable blue emissions. We have noticed that the all-inorganic metal halide double perovskite material Cs_2_ZrCl_6_ has received widespread attention recently, and this material can produce blue-light emission under ultraviolet light excitation [25,26]. Research results show that this blue luminescence comes from STEs generated by the strong electron–phonon coupling effect of the [ZrCl_6_]^2-^ inorganic octahedron in Cs_2_ZrCl_6_ [26]. Therefore, Zr^4+^ ions can be incorporated into host materials as optically active ions to achieve blue-light emission.

Herein, we used nontoxic and stable all-inorganic Cs_2_ZnCl_4_ as the host and Zr^4+^ ions as the dopant to design a zero-dimensional metal halide Zr^4+^:Cs_2_ZnCl_4_ with efficient blue emission. By introducing Zr^4+^ into non-luminescent Cs_2_ZnCl_4_, efficient blue-light emission (λ_em_ = 456 nm under 260 nm UV excitation) was achieved with a PLQY as high as 89.67%. Detailed optical characterization including photoluminescence (PL) decay lifetime and temperature-dependent PL spectra demonstrated that the bright blue emission in Zr^4+^:Cs_2_ZnCl_4_ originated from self-trapped exciton (STE) emission. We believe that this work can provide a reference for the future design and synthesis of low-toxic metal halide materials with efficient blue emission properties.

## 2. Results and Discussion

### 2.1. Structure and Morphology

**Figure 1:**
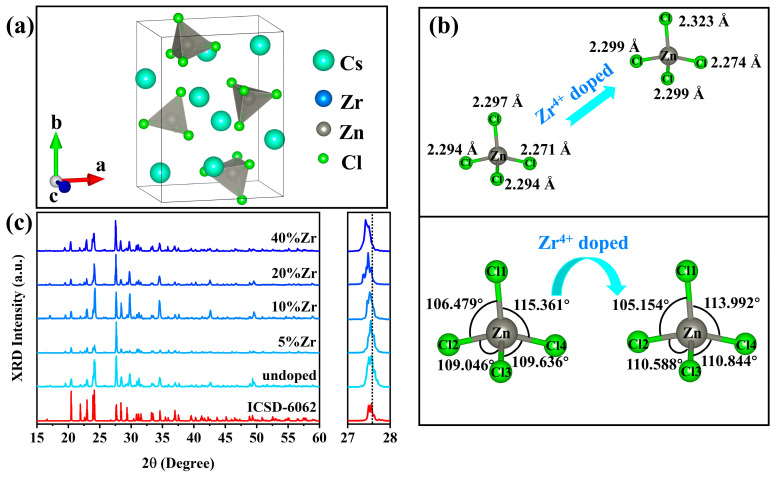
(**a**) The crystal structure of Cs_2_ZnCl_4_. (**b**) The bond length of Zn-Cl and the bond angle of Cl-Zn-Cl in a [ZnCl_4_]^2−^ tetrahedron before and after Zr^4+^ doping. (**c**) The XRD patterns of Cs_2_ZnCl_4_ with different Zr^4+^ feed ratios.

Pure Cs_2_ZnCl_4_ and Zr^4+^-doped Cs_2_ZnCl_4_ crystal samples were prepared by the solvothermal method using cesium chloride, zinc chloride, and zirconium chloride. Crystallographic data indicated that Cs_2_ZnCl_4_ belonged to the orthorhombic *Pnma* space group and had a typical 0D structure. In each [ZnCl_4_]^2−^ tetrahedron, one Zn^2+^ ion occupied the central position and four Cl^−^ ions were connected to it to form a highly symmetrical tetrahedral geometry, as shown in Figure 1a. Each [ZnCl_4_]^2−^ tetrahedron was distributed in isolation and the distance between two adjacent tetrahedrons was 6.6 Å (calculated based on the Zn^2+^ ion distance). The tetrahedrons exhibited a typical 0D structure at the molecular level. Cs^+^ ions served as a supporting framework and charge balance in the structure. As shown in the upper part of Figure 1b, the bond length of the Zn-Cl bond was between 2.271 Å and 2.297 Å in each [ZnCl_4_]^2−^ tetrahedron. The bond length range of the Zn-Cl bond in the [ZnCl_4_]^2−^ tetrahedron changed to 2.274 Å~2.323 Å after Zr^4+^ ions were introduced into the lattice of Cs_2_ZnCl_4_. Compared with undoped Cs_2_ZnCl_4_, the introduction of Zr^4+^ ions significantly increased the bond length of the Zn-Cl bond, which showed that the tetrahedral distortion in the doped system was greater than the undoped system. In addition, the bond angles of the four Cl-Zn-Cl also changed significantly after Zr^4+^ doping. In the undoped system, the bond angles of Cl1-Zn-Cl2, Cl2-Zn-Cl3, Cl3-Zn-Cl4, and Cl1-Zn-Cl4 were 106.479°, 109.046°, 109.636°, and 115.361°, respectively. After the introduction of Zr ions, the Cl1-Zn-Cl2 and Cl1-Zn-Cl4 bond angles decreased, while the Cl2-Zn-Cl3 and Cl3-Zn-Cl4 bond angles increased, as shown in the lower part of Figure 1b. The significant structural differences between the undoped system and the doped system also provide evidence that Zr^4+^ ions replaced a small part of the Zn^2+^ ions lattice. At the same time, this significant difference also showed the stronger distortion of the crystal lattice after Zr^4+^ doping, and easily generated the strong electron–phonon coupling effects under excitation, which is an intrinsic condition for efficient light emission [27,28,29].

The powder x-ray diffraction (PXRD) patterns of Cs_2_ZnCl_4_ crystal samples at different Zr^2+^ doping concentrations are shown in Figure 1c. Each pattern was in good agreement with the standard diffraction pattern of Cs_2_ZnCl_4_ (ISCD-6062) and no redundant impurity diffraction peaks appeared. This result shows that all samples were successfully synthesized and no impurity phase was produced. The uniformity of these patterns also confirms that the doping of Zr^4+^ did not change the original crystal structure of the Cs_2_ZnCl_4_ host material. At the same time, the diffraction pattern of the sample showed a tendency to move to a lower angle as the Zr^4+^ feed ratio continued to increase, which confirms the successful doping of Zr^4+^ ions. Because larger Zr^2+^ ions (ionic radius: 0.6 Å for Zn^2+^ and 0.72 Å for Zr^4+^) were incorporated into the Cs_2_ZnCl_4_ lattice, the lattice expanded, leading to the movement of the XRD diffraction peak to a lower angle [30,31]. The elemental composition of 20%Zr^4+^:Cs_2_ZnCl_4_ was strictly characterized qualitatively and quantitatively. Energy spectroscopy (EDS) elemental mapping of the Zr^4+^:Cs_2_ZnCl_4_ crystal showed that Cs, Zn, Cl, and Zr elements were uniformly distributed (Figure S1a), indicating that Zr^4+^ ions were uniformly doped in the Cs_2_ZnCl_4_ lattice, with a high phase purity. The measurement results showed that the atomic ratio of Cs:Zn:Zr:Cl was 33.55:11.26:0.39:54.81 while the feed ratio of Zr^4+^ ions was 20% (Figure S1b). The results of the elemental analysis showed that when the feed ratio of the Zr^4+^ ions in the experiment was 20%, the actual atomic percentage entering the Cs_2_ZnCl_4_ lattice was only 3.35%. This may have been due to the larger radius and higher valence of Zr^4+^ ions, which could not easily replace the lattice position of Zn^2+^.

### 2.2. Optical Properties

**Figure 2:**
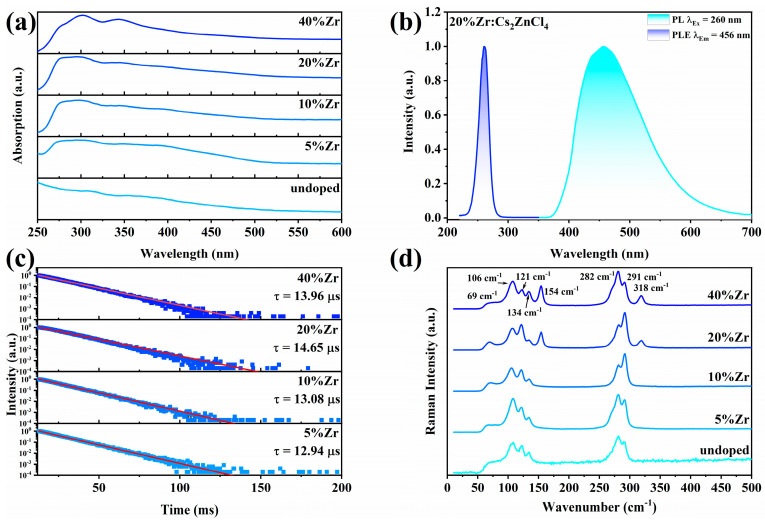
(**a**) Absorption spectra of Cs_2_ZnCl_4_ with different Zr^4+^ feed ratios. (**b**) Normalized PLE (with the monitoring wavelength at 457 nm) and PL (under the excitation wavelength at 260 nm) spectra of 20%Zr^4+^:Cs_2_ZnCl_4_. (**c**) PL lifetime of Cs_2_ZnCl_4_ with different Zr^4+^ feed ratios monitored at 260 nm. (**d**) Raman spectra of Cs_2_ZnCl_4_ with different Zr^4+^ feed ratios under the excitation of a 630 nm laser.

In order to study the optical properties of Zr^4+^:Cs_2_ZnCl_4_, the absorption spectra of Cs_2_ZnCl_4_ at different Zr^4+^ doping ratios were first collected, as shown in Figure 2a. The absorption spectrum of the undoped sample showed that absorption intensity increased slowly as the wavelength decreased and no obvious absorption band appeared in the range from 250 nm to 600 nm. With the introduction of Zr^4+^ ions, two new absorption bands appeared at 300 nm and 350 nm. The intensity of these two absorption bands increased with the increase in the Zr^4+^ ion doping ratio. There is no doubt that the optical transition process corresponding to these two absorption bands was related to the exciton characteristics generated by structural distortion, which originated from the incorporation of Zr^4+^ ions [32,33,34].

Under the excitation of 260 nm UV light, Zr^4+^:Cs_2_ZnCl_4_ exhibited bright broadband blue emission in the range of 370 nm to 700 nm with a PL peak position at 456 nm, Stokes shift of 197 nm, and a full-width half maximum (FWHM) of 119.86 nm. The CIE coordinates of Zr^4+^:Cs_2_ZnCl_4_ and the optical photograph of the sample under 254 nm UV excitation are shown in Figure S2a. The PL intensity changed significantly due to the concentration quenching effect, showing an increase first and then a downward trend with the increase in the feeding ratios of Zr^4+^ (Figure S2b). When the Zr^4+^ feeding ratio was 20%, the PL intensity reached the maximum. In order to investigate the potential photophysical mechanism of these Zr^4+^ doped samples, the PLE spectra of Zr^4+^:Cs_2_ZnCl_4_ at different emission wavelengths and the PL spectra at different excitation wavelengths were collected. Figure S3a shows that the PLE spectra at different emission wavelengths all had similar profiles, which excluded the possibility that the blue emission came from other defects or impurities in the crystal. Figure S3b shows that the PL spectra of the samples obtained with different excitation wavelengths also had the same shape, indicating that this blue emission came from the same excited state relaxation.

Figure 2c shows the PL lifetime decay curve of Cs_2_ZnCl_4_ under different Zr^4+^ doping ratios. The lifetime curves of all samples could be fitted using single exponential decay. All these samples exhibited decay lifetimes in the microsecond range. PL decay lifetime in the order of microseconds is a typical feature of STE emission, and the lifetime of this emission is usually longer than the lifetime of band-edge free excitons [27,35]. As the Zr^4+^ doping amount gradually increased, the PL lifetime of these samples showed a trend of first increasing and then decreasing, with the longest lifetime at 20% doping amount, which was similar to the change trend of the PL spectrum (Figure S2b). The decrease in lifetime at high doping ratios was due to the concentration quenching effect of high concentrations of Zr^4+^ ions because high concentrations of Zr^4+^ ions will generate more non-radiative recombination paths to quench the emission. Characteristics such as broadband emission and long lifetime in the order of microseconds indicated that the bright blue emission in Zr^4+^:Cs_2_ZnCl_4_ came from STE emission caused by strong electron–phonon coupling inside the crystal lattice. This strong electron–phonon coupling effect exists widely in “soft” lattice materials with less structural rigidity [35,36]. The strong electron–phonon coupling effect will cause elastic deformation of the lattice in this “soft” lattice structure, thereby binding the photogenerated excitons to certain specific positions on the lattice to form STEs. This state has a lower energy level than other excited states and, therefore, can exist stably. These excitons are bound to specific positions in the crystal lattice, and can only radiate photons outward through radiative recombination to produce emission. In addition, the sample obtained a high PLQY of 89.67% under 260 nm ultraviolet light excitation when the Zr^4+^ ion doping amount was 20%. This indicates that Zr^4+^:Cs_2_ZnCl_4_ has certain applications in the field of solid-state lighting and display (Figure S4).

In order to explore the electron–phonon coupling effect inside the sample, a 633 nm continuous laser was used as the excitation source to collect the Raman spectra of Cs_2_ZnCl_4_ under different Zr^2+^ doping ratios, as shown in Figure 2d. In all samples, six Raman peaks could be observed in the low wavenumber region (<500 cm^−1^). These were located at 69 cm^−1^, 106 cm^−1^, 121 cm^−1^, 134 cm^−1^, 282 cm^−1^, and 291 cm^−1^, respectively. In addition, new Raman peaks appeared near 154 cm^−1^ and 318 cm^−1^ when the Zr^4+^ feed ratios were 20% and 40%, which may have been caused by high-concentration Zr^4+^ doping. It is worth noting that the Raman peak at 282 cm^−1^ was the double frequency mode of the 134 cm^−1^ Raman peak. This double frequency mode indicated that there was a strong electron–phonon coupling in Zr^4+^:Cs_2_ZnCl_4_, which was very conducive to the formation of STE [37]. This result further proves that the broadband blue emission of Zr^4+^:Cs_2_ZnCl_4_ was a typical STE emission, which came from the strong electron–phonon coupling effect caused by the structure distortion.

**Figure 3:**
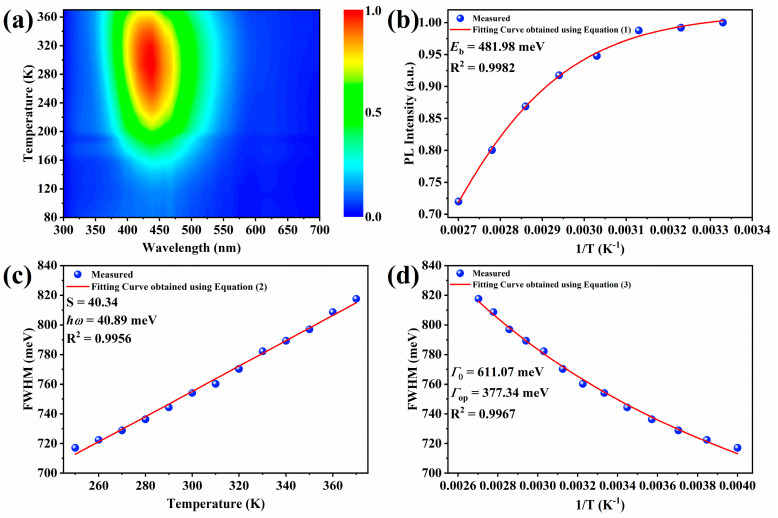
(**a**) The map of the PL intensity of Zr^4+^:Cs2ZnCl4 plotted with temperature T and emission wavelength λ at the temperature range of 80–360 K. (**b**) Fitting results of exciton binding energy *E*_b_. (**c**) Fitting results of Huang–Rhys factor *S*. (**d**) Fitting results of electron–optical-phonon coupling energy.

To further explore the intrinsic photophysical properties in Zr^4+^:Cs_2_ZnCl_4_, the temperature-dependent PL spectrum of Zr^4+^:Cs_2_ZnCl_4_ was measured. Figure 3a shows the map of the PL intensity of Zr^4+^:Cs2ZnCl4 plotted with temperature T and emission wavelength λ at the temperature range of 80–360 K under 260 nm UV excitation. The color scale represents the PL intensity, and the color change from blue to red represents the enhancement of the PL intensity from the minimum to the maximum at the temperature range of 80 K–300 K. Zr^4+^:Cs_2_ZnCl_4_ exhibited a single emission band (λ_em_ = 456 nm) at all temperatures and additional emission bands appeared with temperature changes. In the temperature range of 80 K–150 K, the PL intensity of Zr^4+^:Cs_4_ZnCl_4_ was too weak to show the obvious emission signal. When the temperature was higher than 150 K, the PL intensity of Zr^4+^:Cs_4_ZnCl_4_ began to increase significantly with the increase in temperature and was strongest at 300 K. As the temperature increased further, PL intensity of Zr^4+^:Cs_4_ZnCl_4_ weakened. According to previous research, the formation of STEs requires the participation of a certain intensity of lattice vibration [27,32,35]. In Zr^4+^:Cs_2_ZnCl_4_, the lattice vibration intensity required to generate STEs was stronger. The number of STEs formed was limited and effective emission could not be produced, due to the lattice vibration being significantly suppressed at low temperatures. The enhanced lattice vibration intensity promoted the formation of STEs when the temperature gradually increased, resulting in enhanced PL intensity. Finally, the promotion effect of lattice vibration on emission reached the maximum at 300 K, and the PL intensity was the highest. With further temperature increase, the strong lattice vibration will no longer promote emission but will produce a quenching effect. Therefore, the PL intensity of Zr^4+^:Cs_2_ZnCl_4_ began to decrease when the temperature was higher than 300 K.

In addition, several key physical parameters, such as the Huang–Rhys factor (*S*), the exciton binding energy (*E*_b_), and the electron–optical-phonon coupling energy (*Γ*_op_) were obtained by fitting the temperature-dependent PL spectrum. These physical parameters help to understand the underlying photophysical mechanism in Zr^4+^:Cs_2_ZnCl_4_. First, the exciton binding energy of the Zr^4+^:Cs_2_ZnCl_4_ was fitted using the following formula [38]:

(1) $$I(T)\;=\frac{I_{0}}{\left(1+Ae^{\frac{-E_{b}}{k_{b}T}}\right)}$$

where *I*_0_ is the PL intensity at 0 K, *I*(*T*) is the PL intensity at temperature *T* K, *k_b_* is the Boltzmann constant, and *A* is a constant. The fitting results, as given in Figure 3b, showed that the *E_b_* of the Zr^4+^:Cs_2_ZnCl_4_ was 481.98 meV. Such a large exciton binding energy indicated that these STEs required high energy to generate, which was consistent with the optical behavior at different temperatures. At the same time, the larger the exciton binding energy, the more stable the photogenerated excitons and the higher the dissociation temperature, thereby ensuring that the emission had excellent thermal stability and resistance to thermal quenching.

The Huang–Rhys factor (*S*) of the sample was obtained to investigate the electron–phonon coupling effect by fitting using the following formula [39]:

(2) $$\mathrm{FWHM}\left(T\right)=2.36\sqrt{S}\hbar \omega_{\mathrm{phonon}}\sqrt{\coth\frac{\hbar \omega_{\mathrm{phonon}}}{2k_{b}T}}$$

where *ω*_phonon_ is the phonon frequency, *T* is the temperature, FWHM is the full-width half maximum of the PL spectra, and *k_b_* is the Boltzmann constant. The fitting results are shown in Figure 3c. The Huang–Rhys factor *S* of 40.34 meant that Zr^4+^:Cs_2_ZnCl_4_ had strong electron–phonon coupling. At the same time, the phonon energy ℏ*ω*_phonon_ obtained by fitting was 40.89 meV, which was very close to the vibration energy of the 291 cm^−1^ Raman peak in the Raman spectrum (~36.08 meV). This indicates that the 291 cm^−1^ Raman peak mainly participated in the formation of STEs in Zr^4+^:Cs_2_ZnCl_4_. The large *S* value, broadband emission, and large Stokes shift (197 nm) indicated that the blue emission of Zr^4+^:Cs_2_ZnCl_4_ originated from self-trapped excitons generated by strong Jahn–Teller distortion. The Toyokawa equation was used to fit the change in PL spectrum FWHM with temperature to further investigate the electron–phonon coupling effect in the Zr^4+^:Cs_2_ZnCl_4_ [40]:

(3) $$\Gamma (T)\;=\Gamma_{0}+\frac{\Gamma_{\mathrm{op}}}{e^{\frac{\hbar \omega_{op}}{k_{b}T}}-1}$$

where *Γ*_0_ is the FWHM of PL spectra at 0 K, *Γ*_op_ is the electron–optical-phonon coupling energy, ℏ*ω*_op_ is the energy of long optical phono, and *k_b_* is the Boltzmann constant. ℏ*ω*_op_ was obtained by fitting the Huang–Rhys factor *S*, which was 40.89. As shown in Figure 3d, the fitted *Γ*_0_ and *Γ*_op_ were 611.07 meV and 377.34 meV, respectively. Such a large electron–optical-phonon coupling energy *Γ*_0_ indicates that there was a strong electron–phonon coupling in the Zr^4+^:Cs_2_ZnCl_4_ structure, which had a crucial impact on the generation of STEs.

### 2.3. Theoretical Calculation and Photophysical Model

**Figure 4:**
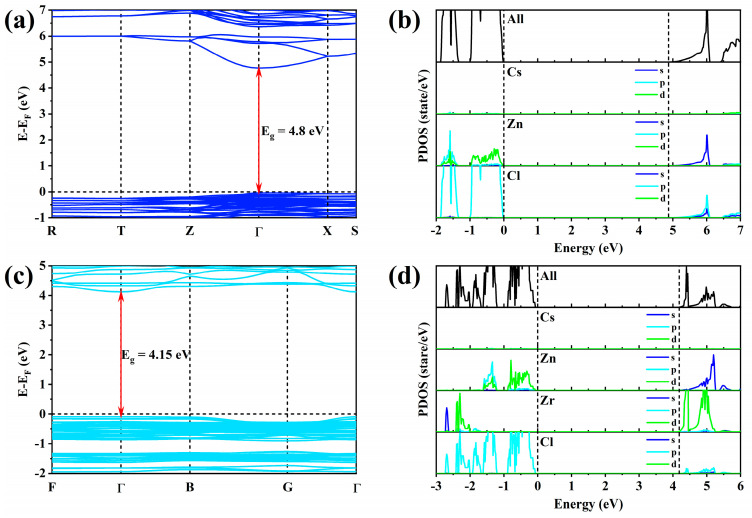
(**a**) Bandgap structure of undoped Cs_2_ZnCl_4_. (**b**) DOS of undoped Cs_2_ZnCl_4_. (**c**) Bandgap structure of Zr^4+^:Cs_2_ZnCl_4_. (**d**) DOS of Zr^4+^:Cs_2_ZnCl_4_.

In order to explore the electronic structure of Zr^4+^:Cs_2_ZnCl_4_ and the intrinsic physical mechanism of efficient blue emission, the bandgap structure and density of state (DOS) of Cs_2_ZnCl_4_ and Zr^4+^:Cs_2_ZnCl_4_ were calculated using first principles. Figure 4a,c show the bandgap structures of Cs_2_ZnCl_4_ and Zr^4+^:Cs_2_ZnCl_4_, respectively. Interestingly, the band edge after doping with Zr^4+^ ions was flatter than that of pure Cs_2_ZnCl_4_, which means that the doping of Zr^4+^ ions enhanced the quantum confinement effect because the flat conduction band and valence band meant small dispersion and large carrier effective mass, which is a typical feature of a strong quantum confinement effect [41]. The strong quantum confinement effect was very important for the highly localized exciton formation. Once excitons were formed under excitation, they would be confined to isolated inorganic polyhedrons due to the unique quantum confinement effect of the zero-dimensional structure. There was no interaction due to the large distance between adjacent polyhedrals, leading to highly efficient luminescence in Zr^4+^:Cs_2_ZnCl_4_. Figure 4b,d show the DOS of Cs_2_ZnCl_4_ and Zr^4+^:Cs_2_ZnCl_4_, respectively. As shown in Figure 4b, the valence band maximum (VBM) of Cs_2_ZnCl_4_ was mainly composed of Zn-3d and Cl-3p orbitals, while Zn-4s, Cl-3p, and Cl-3s orbitals together constituted the conduction band minimum (CBM). The VBM of Zr^4+^:Cs_2_ZnCl_4_ was mainly composed of Zn-3d and Cl-3p orbitals, while the Cl-3p and Zr-4d orbitals together constituted the CBM of Zr^4+^:Cs_2_ZnCl_4_ (Figure 4d). In addition, we also calculated the 3D and 2D electron distribution maps of VBM and CBM in the two compounds, as shown in Figure S5. Through the 3D and 2D electron distribution diagrams of VBM and CBM in pure ((a)–(d)) Cs_2_ZnCl_4_ and ((e)–(h)) Zr^4+^:Cs_2_ZnCl_4_, we can intuitively see that the VBM of Cs_2_ZnCl_4_ was mainly composed of Zn and Cl orbitals, while Zn and Cl orbitals together constituted the CBM. The VBM of Zr^4+^:Cs_2_ZnCl_4_ was mainly composed of Zn and Cl orbitals, while the Zr orbitals constituted the CBM of Zr^4+^:Cs_2_ZnCl_4_. The DOS of Zr^4+^:Cs_2_ZnCl_4_ showed that its bright blue emission was related to the doping of Zr^4+^ ions. Combining the PL spectrum and PL decay lifetime, it can be further confirmed that this blue emission originated from self-trapped excitons generated by lattice distortion caused by Zr^4+^ ion doping. The ultra-high PLQY of close to 100% in Zr^4+^:Cs_2_ZnCl_4_ could be assigned to the highly localized exciton distribution in the structure, which benefited from the strong quantum confinement effect unique to the zero-dimensional structure.

**Figure 5:**
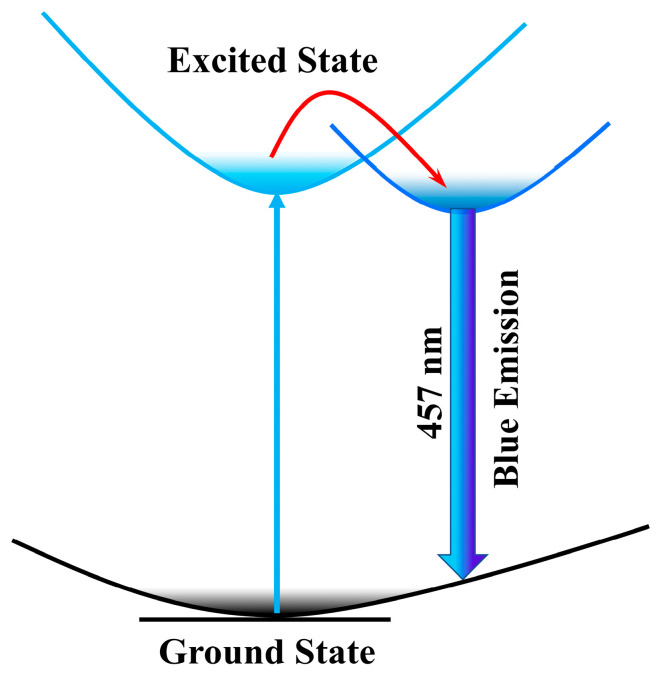
Schematic diagram of the photophysical model of Zr^4+^:Cs_2_ZnCl_4_.

As shown in Figure 5, ground state electrons absorbed energy and transited to the excited state under UV excitation. Due to the soft lattice properties of Zr^4+^:Cs_2_ZnCl_4_, the lattice deformed rapidly after being excited and bound the excited electrons to specific sites in the lattice. The bound electrons then radiated photons outward through radiative recombination and produced bright blue-light emission.

## 3. Conclusions

In summary, in this work, we report an all-inorganic zero-dimensional metal halide Zr^4+^:Cs_2_ZnCl_4_ with efficient broadband blue emission. Under the excitation of 260 nm UV light, Zr^4+^:Cs_2_ZnCl_4_ exhibited broadband blue emission at 457 nm, with a PLQY of up to 89.67%. Combining the room- and low-temperature PL spectra with the PL decay lifetime, confirmed that the broadband blue emission in Zr^4+^:Cs_2_ZnCl_4_ came from self-trapped excitons generated by structural distortion after Zr^4+^ ion doping. First-principles calculation results showed that the efficient emission came from the strong quantum confinement effect generated by the unique zero-dimensional structure of Cs_2_ZnCl_4_. We believe this work can provide a reference for the future design and synthesis of low-dimensional metal halide materials with high luminescence efficiency.

## Data Availability

Data are contained within the article and Supplementary Materials.

## Supplementary Materials

The following supporting information can be downloaded at: https://www.mdpi.com/article/10.3390/molecules29071651/s1, Experimental and computational details and supplementary figures (PDF). Ref. [42] is cited in Supplementary Materials.
