# Supplementary material for: Boosting Blue Self-Trapped Exciton Emission in All-Inorganic Zero-Dimensional Metal Halide Cs2ZnCl4 via Zirconium (IV) Doping
Source: Molecules. 2024 Apr 6;29(7):1651. doi: 10.3390/molecules29071651 (PMC11013416; doi:10.3390/molecules29071651)
Supplement: Supplementary file 1 [file molecules-29-01651-s001.zip › molecules-2946860-supplementary.pdf]

Supporting Information

Boosting Blue Self-Trapped Exciton Emission in All-Inorganic Zero-Dimensional Metal Halide Cs_2ZnCl_4 via Zirconium (IV) Doping

Ye Tian [1], Qilin Wei [2,*], Lian Duan [3] and Chengyu Peng [3,*]

[1] School of Semiconductors and Physics, North University of China, Taiyuan 030051, China; 20230127@nuc.edu.cn
[2] School of Chemistry and Chemical Engineering, Shandong University, Jinan 250100, China
[3] Traffic Information Engineering Institute, Guangxi Transport Vocational and Technical College, Nanning 530004, China; duanlianxs@foxmail.com
* Correspondence: qlwei@sdu.edu.cn (Q.W.); chengyu_peng@126.com (C.P.)

Experimental method and characterization

Chemicals

Cesium chloride (CsCl; AR), Zinc chloride ($ZnCl_2$; 99%), Zirconium chloride ($ZrCl_4$, 99.5%) are all purchased from Macklin. Hydrochloric acid (HCl; ChengDu Chron Chemicals Co,.Ltd, AR, 37 wt% in water), ethanol (EtOH; Guangdong Guanghua Sci-Tech Co,.Ltd, AR) were used without any purification.

Synthesis of Zr^{4+}:Cs_2ZnCl_4 crystals with Zr^{4+} feed ratio = 0, 5%, 10%, 20%, 40%)

Zr^{4+}:Cs_2ZnCl_4 crystals were synthesis by a solvent thermal method. 2mmol CsCl, 1 mmol $ZnCl_2$ and a certain amount of $ZrCl_4$ (the amount of $ZrCl_4$ was decided by the relative mole percent of Zn), 5 mL HCl was added in the 25 mL stainless steel autoclave. The mixture solution was heated at 160 °C for 4 h and then cooled slowly to room temperature. The precipitated Zr^{4+}:Cs_2ZnCl_4 crystals were washed with ethanol and were dried at 80 °C for 8 h.

Material characterization

A SMARTLAB 3KW X-ray diffractometer with Cu Kα radiation (λ= 1.54059 Å) was used to collect the powder X-ray diffraction data in the 2θ range of 15°-60°. The scanning electron microscopy (SEM, Hitachi SU8020) was used to observe the morphology. The energy-dispersive spectrometry (EDS, Oxford X-Max Aztec) was used to collect the element composition and distribution. The Raman spectrum were characterized by WITec alpha300R Raman fluorescence spectrometer with a 633 nm laser as an excitation source. The photoluminescence (PL), PL

excitation (PLE) spectrum, time-resolved photoluminescence (TRPL), photoluminescence quantum yields (PLQYs), temperature-dependent PL spectra, and temperature-dependent PL lifetime were obtained on the Edinburgh FLS-1000 spectrofluorometer. The Lambda 750 ultraviolet-visible spectrophotometer was used to measure the absorption spectrum.

Computational Details

The projector-augmented wave method is used to calculate the band structure, as implemented in the Quantum Espresso soft[1]. The generalized gradient approximation of the Perdew-burke-Ernzerhof parameterization is used for the exchange and correlation functional. The kinetic energy cutoff of 500 eV and a 4×4×4 Monkhorst-Pack k-mesh for the wavefunction basis set is used. The energy convergence criterion is set as 1.0×10^{-6} eV for structure relaxations.

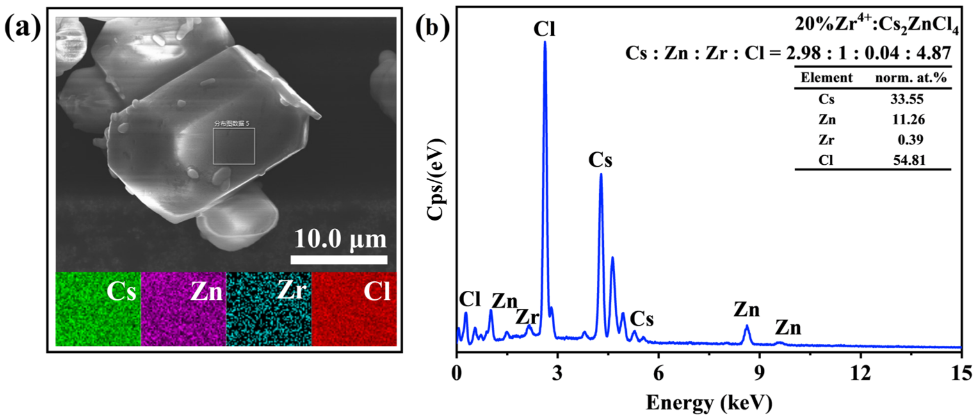

Figure S1. (a) The SEM image and element distribution of Zr^{4+}:Cs_2ZnCl_4. (b) The EDS spectrum and elemental analysis of Zr^{4+}:Cs_2ZnCl_4.

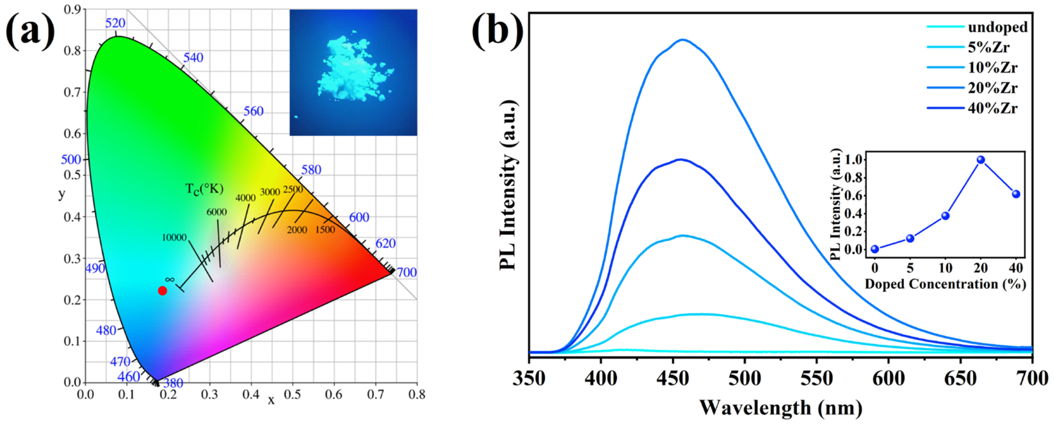

Figure S2. (a) The CIE color coordinates of blue emission in Zr^{4+}:Cs_2ZnCl_4. Insert: the optical photograph of Zr^{4+}:Cs_2ZnCl_4 microcrystals under 254 nm UV light. (b) the PL spectra of Cs_2ZnCl_4

with different Zr^{4+} feed ratio. Insert: the PL intensity of Cs_2ZnCl_4 with different Zr^{4+} feed ratio.

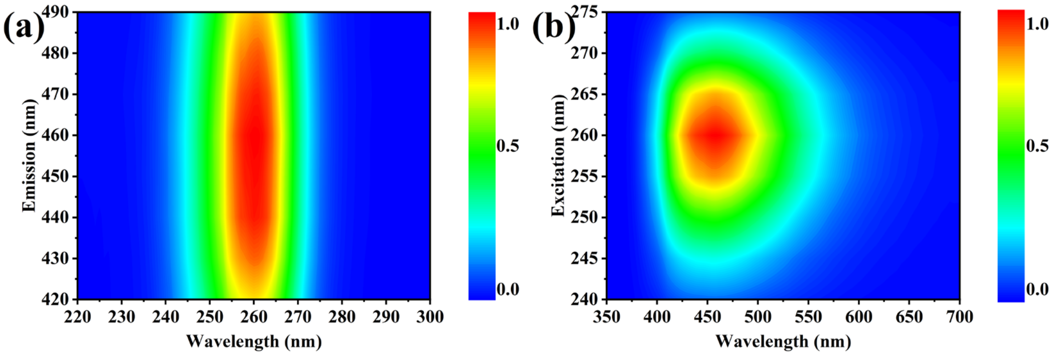

Figure S3. (a) The PLE spectra of Zr^{4+}:Cs_2ZnCl_4 under different emission wavelength. (b) The PL spectra of Zr^{4+}:Cs_2ZnCl_4 under different excitation wavelength.

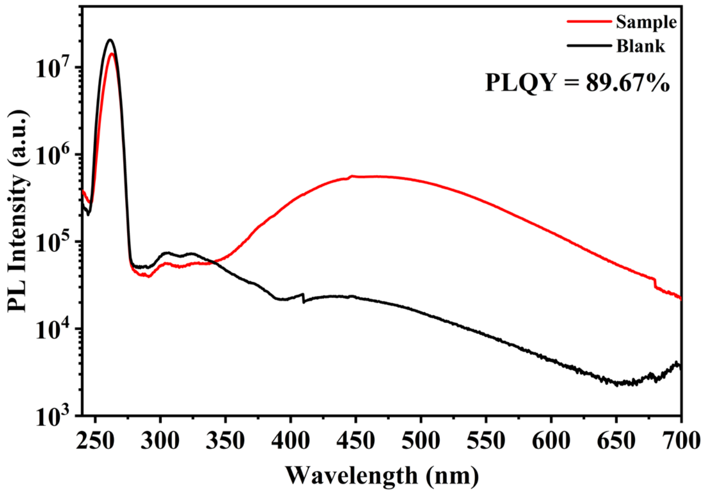

Figure S4. The PLQY of 20%Zr^{4+}:Cs_2ZnCl_4.

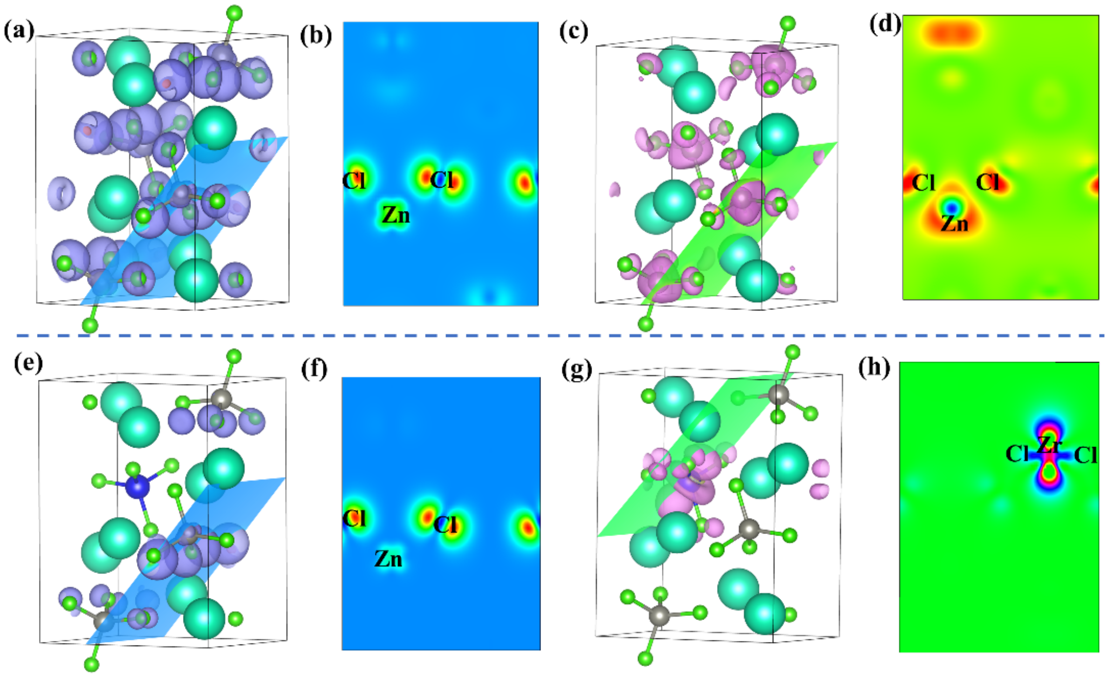

Figure S5. 3D and 2D Electron distribution profiles of VBM and CBM in pure (a)-(d) Cs_2ZnCl_4 and (e)-(h) Zr^{4+}:Cs_2ZnCl_4, respectively.

Refernce

1. Giannozzi, P.; Baroni, S.; Bonini, N.; Calandra, M.; Car, R.; Cavazzoni, C.; Ceresoli, D.; Chiarotti, G. L.; Cococcioni, M.; Dabo, I., QUANTUM ESPRESSO: A modular and Ppen-Source Software Project for Quantum Simulations of Materials. *J. Phys.: Condens. Matter* **2009,** 21, (39), 395502.
